# ADAR1 has an oncogenic function and can be a prognostic factor in cervical cancer

**DOI:** 10.1038/s41598-023-30452-y

**Published:** 2023-03-23

**Authors:** Keiichiro Nakamura, Kunitoshi Shigeyasu, Kazuhiro Okamoto, Hirofumi Matsuoka, Hisashi Masuyama

**Affiliations:** 1 Department of Obstetrics and Gynecology, Okayama University Graduate School of Medicine, Dentistry and Pharmaceutical Sciences, 2-5-1 Shikata-Cho, Kita-Ku, Okayama, 700-8558 Japan; 2 Department of Gastroenterological Surgery, Okayama University Graduate School of Medicine, Dentistry and Pharmaceutical Sciences, 2-5-1 Shikata-Cho, Kita-Ku, Okayama, 700-8558 Japan

**Keywords:** Cancer, Medical research, Molecular medicine, Oncology

## Abstract

Adenosine deaminase acting on RNA 1 (ADAR1), a recently described epigenetic modifier, is believed to play a critical oncogenic role in human cancers. However, its functional role and clinical significance in cervical cancer (CC) remain unclear. ADAR1 knockdown was performed to investigate its oncogenic functions in SiHa (HPV16), HeLa (HPV18), and Yumoto (non-HPV) CC cell lines. Cytoplasmic and nuclear ADAR1 expression were examined to clarify their correlation with clinicopathological parameters and prognosis in patients with CC. This resulted in increased apoptosis and necroptosis in HPV16 -type SiHa, HPV18-type HeLa, and non-HPV-type Yumoto CC cell lines. Progression-free survival (PFS) rates of patients exhibiting high cytoplasmic and nuclear ADAR1 expression were poorer than those in the other groups (*P* = 0.016). Multivariate analysis indicated that the combination of higher cytoplasmic and nuclear ADAR1 expression was an independent predictor of prognosis in patients with CC (*P* = 0.017). ADAR1 could be a potential therapeutic target for HPV-positive or HPV-negative CC. The combination of cytoplasmic and nuclear ADAR1 comprises a better prognostic factor for CC.

It is estimated that cervical cancer (CC) will cause 14,100 new cases and 4,280 new deaths in the United States in 2022^1^. CC is caused by persistent infections with carcinogenic human papillomavirus (HPV). This disease is caused mainly by persistent HPV infection (types 16 and 18), accounting for approximately 70% of all CCs^2,3^. Similar to that for other carcinomas, The Cancer Genome Atlas (TCGA) provides an extensive molecular characterization based on patients with CC but not a practical genetic analysis^4^. Therefore, a better understanding of the molecular mechanisms underlying the progression of CC is needed to gain insight into novel therapeutic targets.

Recent studies on cancer mechanisms have focused on the mechanism of DNA imbalances, but these have not led to effective genetic analyses and treatments. Tumorigenesis might be caused by RNA transcription, in which an RNA editing enzyme plays an important role. RNA editing is a recently identified epigenetic mechanism that regulates the post-transcriptional activity of key oncogenes by altering their amino acid sequences, leading to changes in their oncogenic functions^5^. One such RNA editing process, wherein the conversion of adenosine (A) to inosine (I) in primary RNA transcripts (A-to-I editing) is mediated by a family of adenosine deaminases acting on RNA (ADAR), leads to transcriptome diversification in human cells^6^. Since recent reports have focused on it role in the direct regulation of cell death, such as apoptosis, aside from RNA editing functions^7^. we decide to focus on the regulation of cell death by ADAR1 in this paper, without mentioning A-to-I editing. This family includes three enzymes, ADAR1, ADAR2, and ADAR3. ADAR1 and ADAR2 are ubiquitously expressed, and unlike brain-specific ADAR3, they exhibit catalytic activity^8–12^. ADAR1 has two isoforms, the shorter and constitutive ADAR1p110 and the full-length ADAR1 p150^13^. It has been proven that ADAR1 p150 is located in the cytoplasm and nucleus, whereas ADAR1 p110 is mainly expressed in the nucleus^14^. ADAR1p150 can move between the nucleus and cytoplasm as a shuttling protein^15,16^. Both ADAR1p110 and ADAR1p150 suppress interferon (IFN) expression and IFN-mediated antiviral activity^17^. The extended N-terminus of ADAR1 p150, including the additional Zɑ domain, might contribute to these differences. Z-form nucleic acid-binding protein 1 (ZBP1) has a related Zɑ domain that binds Z-RNA and Z-DNA and is induced by IFN^18,19^.

Several studies have reported that ADAR1 is overexpressed in hepatocellular, gastric, colorectal, and endometrial cancers. Moreover, its overexpression is correlated with clinically aggressive behavior and patient survival^20–23^. However, the role of ADAR1 in CC has not yet been investigated in detail. Therefore, this study aimed to explore how ADAR1 could contribute to the malignancy of CC. We further demonstrated how the role of ADAR1 expression in CC can help us gain novel insights into new therapeutic targets.

## Results

### ADAR1 knockdown attenuates ADAR1, ADAR1p110, and ADAR1p150 expression in HPV-positive and HPV-negative CC cell lines

**Figure 1:**
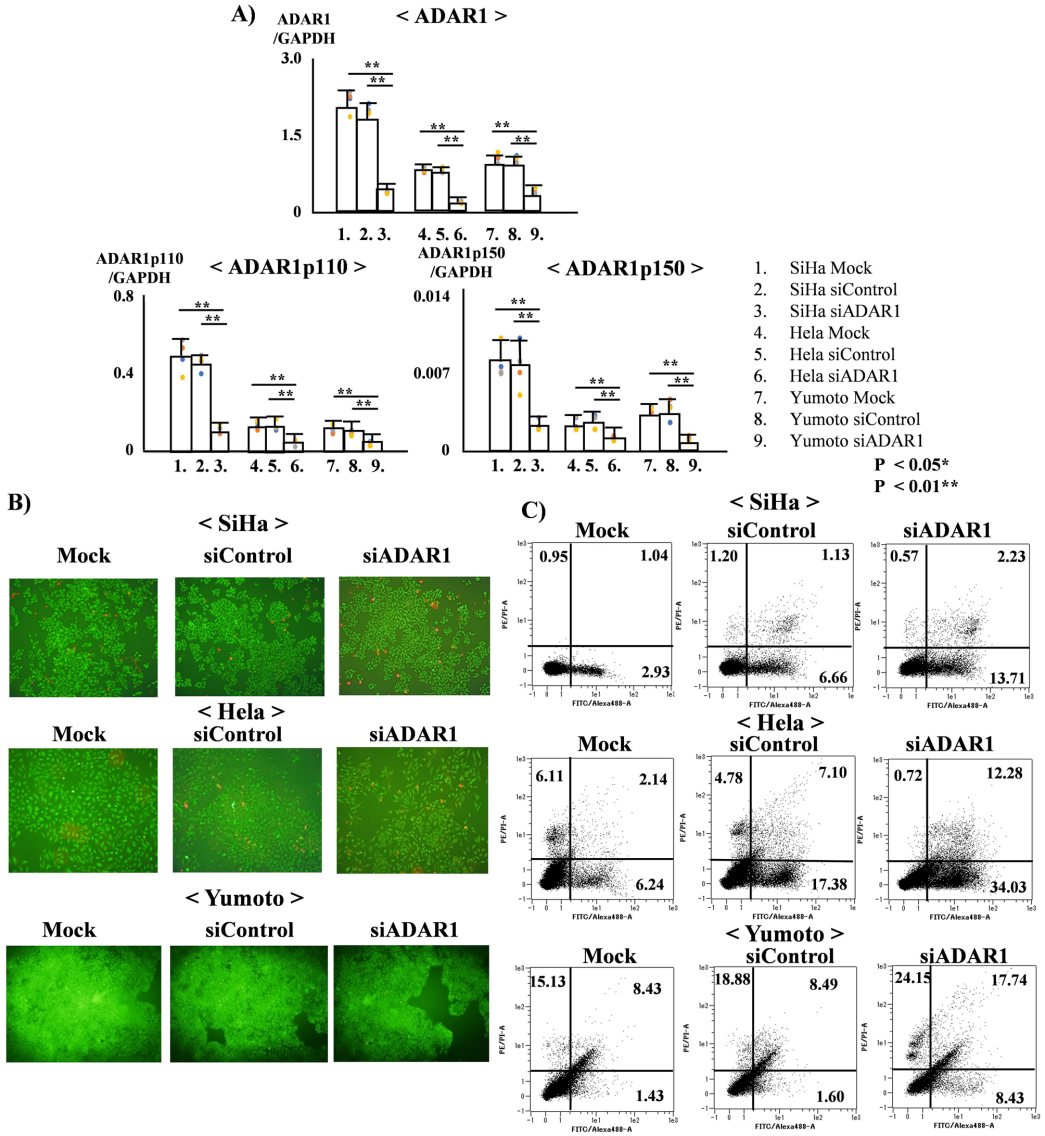
(**A**) Real-time PCR analysis of the ADAR1, ADAR1p110, and ADAR1p150 expression levels after transient transfection with the mock conditions, control siRNA (siCon), or ADAR1 siRNA (siADAR1) into human papilloma virus (HPV) 16-type SiHa, HPV18-type Hela, and non-HPV-type Yumoto cervical cancer (CC) cells for 48 h. The assays were carried out based on quadruplicate transfection experiments. (**B**) Cell viability of HPV16-type SiHa, HPV18-type Hela, and non-HPV-type Yumoto CC cells after transient transfection with the mock, siCon, or siADAR1 was evaluated using a fluorescence microscope for 48 h. (**C**) Representative flow cytometric data for apoptosis and necroptosis of HPV16-type SiHa, HPV18-type Hela, and non-HPV-type Yumoto CC cells after transient transfection with the mock, siCon, and siADAR1 for 48 h.

ADAR1 is strongly associated with viruses, and CC is a highly HPV-related disease. There is a need to understand how the p150 and p110 isoforms are regulated and how they individually contribute to apoptosis and necroptosis through the knockdown of ADAR1 in HPV-positive and HPV-negative CC cell lines. In addition, ADAR1 p110 and p150 have complementary functions. For this reason, when examining the knockdown of ADAR1, it is necessary to ensure that both p110 and 150 are knocked down. The knockdown efficiency of ADAR1 expression was confirmed using real-time PCR (Fig. 1A). ADAR1, ADAR1p110, and ADAR1p150 expression was significantly decreased after transfecting the ADAR1 small-interfering RNA (siRNA) into HPV16-type SiHa, HPV18-type HeLa, and non-HPV type Yumoto cell lines as expected (real-time PCR: ADAR1: all Mock; *P* < 0.001, all Control siRNA; *P* < 0.001; ADAR1p110: Mock; *P* < 0.001, *P* < 0.001, and *P* = 0.001, control siRNA; *P* < 0.001, *P* = 0.001, and *P* = 0.001; ADAR1p150: Mock; *P* < 0.001, *P* = 0.004, and *P* < 0.001, Control siRNA; *P* = 0.002, *P* = 0.006, and *P* < 0.001, Fig. 1A). In the HPV-type and non-HPV-type CC cell lines, ADAR1p110 and ADAR1p150 expression was significantly suppressed through the introduction of ADAR1 siRNA. The number of apoptotic and dead cells increased following the transient transfection of ADAR1 siRNA into HPV16-type SiHa, HPV18-type HeLa cells, and non-HPV-type Yumoto cells (Fig. 1B).

### Apoptosis and necroptosis are increased by the knockdown of ADAR1 in HPV-positive and HPV-negative CC cell lines

The subsequent profiles of apoptosis and necroptosis were obtained after the transient transfection of siADAR1 into HPV16-type SiHa, HPV18-type HeLa, and non-HPV-type Yumoto cells. Representative flow cytometric data revealed that the transient transfection of ADAR1 siRNA for 48 h increased Annexin V-fluorescein isothiocyanate (FITC)- and PI-positive signals. ADAR1 siRNA induced apoptosis, necrosis, and necroptosis in these cancer cells, as assessed by an Annexin V-FITC assay (Fig. 1C). In HPV16-type SiHa, HPV18-type HeLa, and non-HPV-type Yumoto cells, ADAR1 siRNA increased the proportions of cells undergoing early apoptosis, necrosis, and late apoptosis and necroptosis to 13.71%, 34.03%, and 8.43% (early apoptosis: siADAR1), 0.57%, 0.72%, and 24.15% (necrosis: siADAR1), and 2.23%, 12.28%, and 17.74% (late apoptosis and necroptosis: siADAR1), respectively, as compared with proportions in the mock and control groups of 2.93%, 6.24%, and 1.43% (early apoptosis: Mock), 0.95%, 6.11%, and 15.13% (necrosis: Mock), 1.04%, 2.14%, and 8.43% (late apoptosis and necroptosis: Mock), and 6.66%, 17.38%, and 1.60% (early apoptosis: siControl), 1.20%, 4.78%, and 18.88% (necrosis: siControl), and 1.13%, 7.10%, and 8.49% (late apoptosis and necroptosis: siControl), respectively. Therefore, ADAR1 siRNA likely regulates early apoptosis, late apoptosis, and necroptosis rather than necrosis in HPV-positive or HPV-negative CC cell lines.

### Knockdown of ADAR1 in HPV-positive or HPV-negative CC cell lines increases apoptosis and necroptosis

**Figure 2:**
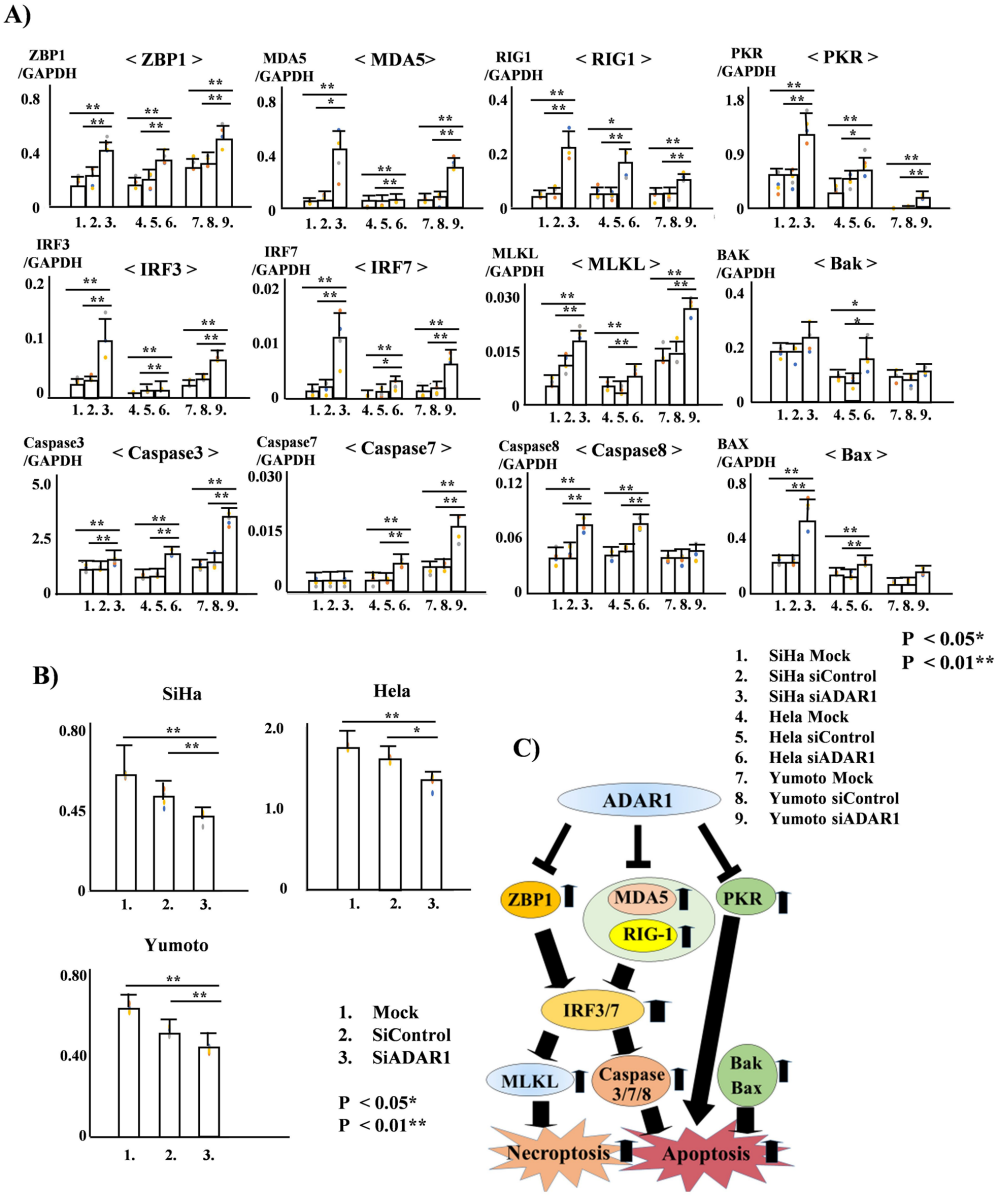
(**A**) Real-time PCR analysis of human papilloma virus (HPV) 16-type SiHa, HPV18-type Hela, and non-HPV-type Yumoto cervical cancer (CC) cells after transient transfection with the mock conditions, control siRNA (siCon), or ADAR1 siRNA (siADAR1) for 48 h. Real-time PCR analysis of ZEBP1, MDA5, RIG-I, PKR, IRF3, IRF7, MLKL, Caspase3, Caspase7, Caspase8, Bak, and Bax expression after transfection with the mock, siCon, or siADAR1 into HPV16-type SiHa, HPV18-type Hela, and non-HPV-type Yumoto CC cells for 48 h. The assays were carried out based on quadruplicate transfection experiments. (**B**) MTS assays on HPV16-type SiHa, HPV18-type Hela, and non-HPV-type Yumoto CC cells after transient transfection with the mock, siCon, and siADAR1 for 48 h. The assays were carried out based on quadruplicate transfection experiments. (**C**) Mechanism of ADAR1 activation in CC.

Based on these previous findings, we hypothesized that ADAR1 might suppress apoptosis and necroptosis in HPV-positive or HPV-negative CC cells by inhibiting the dsRNA-sensing signaling pathway. ADAR1 was shown to suppress innate immunity primarily through retinoic acid-inducible gene-I (RIG-I)-like receptor (RLR)-initiated cytosolic dsRNA-sensing signaling pathways, including differentiation-associated gene 5 (MDA5) and RIG-I^24,25^. RIG-I and MDA-5 induce type I interferon-independent apoptosis^26^. Additionally, the pro-apoptotic functions of ADAR1 are associated with protein kinase R (PKR)^27^. Furthermore, activated ZBP1 can induce necroptosis^28^. Previously, we reported that the suppression of ADAR1 activates the proapoptotic factors MDA5, RIG-I, and PKR, ultimately inducing apoptosis via Bcl-2-family proteins, such as Bak, in endometrial cancer cells^22^. We first examined the effects of ADAR1 knockdown on the expression of ZBP1, MDA5, RIG-I, PKR, interferon regulatory factor 3 (IRF3), and IRF7 in the HPV16-type SiHa, HPV18-type Hela, and non-HPV-type Yumoto CC cell lines. ZBP1, MDA5, RIG-I, PKR, IRF3, and IRF7 expression was significantly increased after the transfection of ADAR1 siRNA into all three cell lines (Fig. 2A). Expression of the IFN-stimulated genes IRF3 and IRF7, which are downstream of MDA5 and RIG-I, was increased after the transfection of ADAR1 into SiHa, Hela, and Yumoto cells (Fig. 2A).

To map the steps involved in apoptosis and necroptosis, we evaluated the activation of mixed lineage kinase domain-like (MLKL), Caspase3, Caspase7, Caspase8, Bak, and Bax. We first examined the effects of ADAR1 knockdown on the expression of MLKL, Caspase3, Caspase7, Caspase8, Bak, and Bax in the HPV16-type SiHa, HPV18-type HeLa, and non-HPV-type Yumoto CC cell lines. MLKL, Caspase3, Caspase8, and Bax expression was significantly increased after the transfection of ADAR1 siRNA into SiHa cells. In Hela cells, MLKL, Caspase3, Caspase7, Caspase8, Bak, and Bax expression was significantly increased after the transfection of ADAR1 siRNA. Additionally, MLKL, Caspase3, and Caspase7 expression was significantly increased after the transfection of ADAR1 siRNA into Yumoto cells (Fig. 2A). These results suggest that the suppression of ADAR1 activates the dsRNA-sensing signaling pathway, which in turn increases the expression of the apoptosis-associated factors MDA5, RIG-I, PKR, Caspase 3, Caspase7, Caspase8, Bax, and Bak and the necroptosis-associated factors ZBP1 and MLKL (Fig. 2C).

### Knockdown of ADAR1 suppresses proliferation in HPV-positive- or HPV-negative-type CC cell lines

We then examined the effects of ADAR1 knockdown on CC cell proliferation. We performed MTS and cell viability assays after transient transfection of the ADAR1 siRNA into HPV16-type SiHa, HPV18-type HeLa, and non-HPV-type Yumoto cells. The numbers of viable cells decreased to 30.2, 24.6, and 31.8% (mock) and 14.3, 17.0, and 17.3% (Control) of control numbers at 48 h after transient transfection of the ADAR1 siRNA into HPV16-type SiHa, HPV18-type HeLa, and non-HPV-type Yumoto cells, respectively (all mock; ADAR1: *P* < 0.001, control siRNA; *P* = 0.011, *P* = 0.001, and *P* < 0.001, Fig. 2B). Therefore, the knockdown of ADAR1 suppressed the proliferation of HPV-positive and HPV-negative CC cell lines.

### Examination of cytoplasmic and nuclear ADAR1 expression to investigate their relationship with various clinicopathological parameters in patients with CC

**Figure 3:**
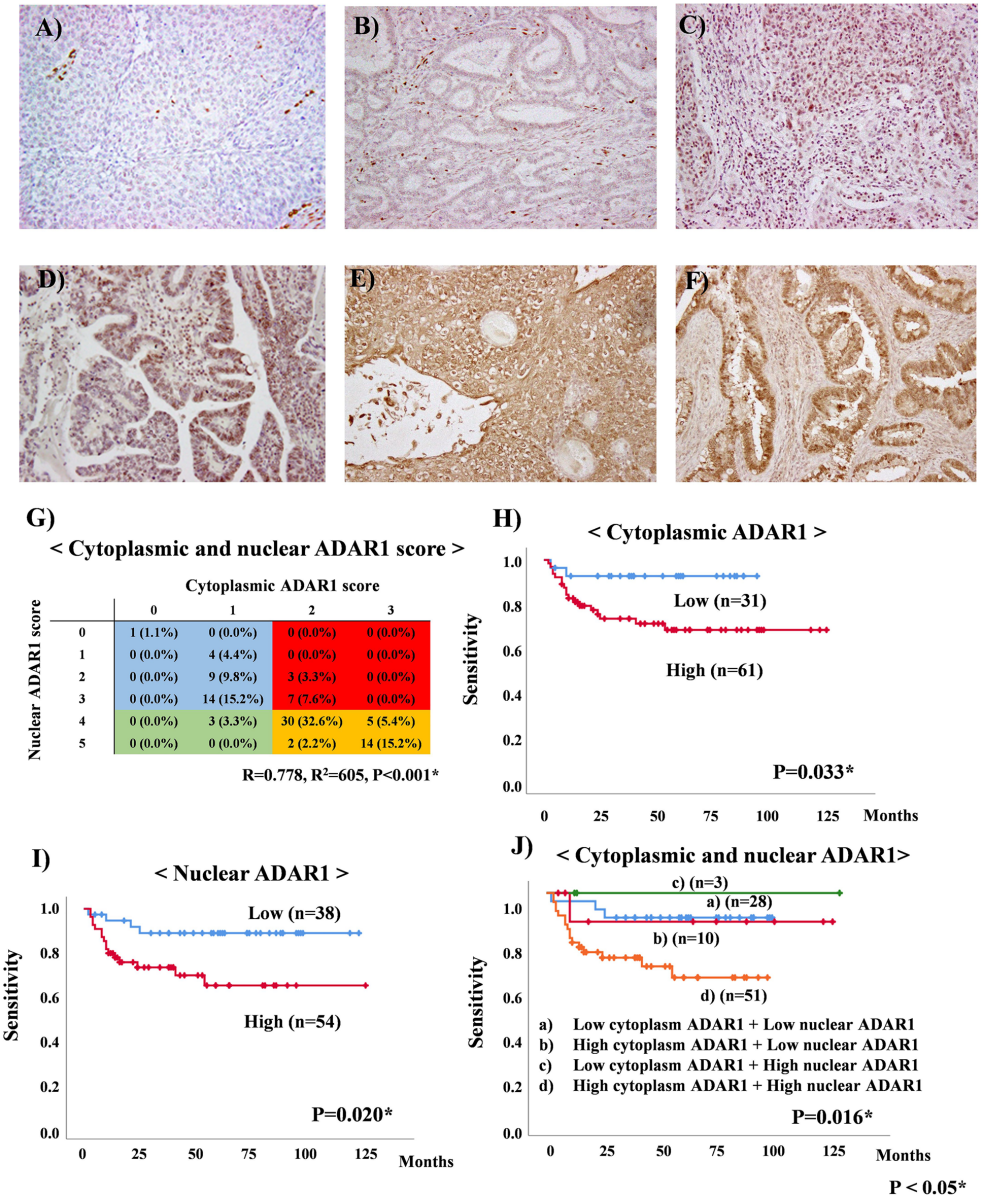
Representative images of cytoplasmic and nuclear ADAR1 expression based on immunohistochemistry. (**A**) Cytoplasmic ADAR1 score (0) and nuclear ADAR1 score (0); (**B**) cytoplasmic ADAR1 score (1) and nuclear ADAR1 score (1); (**C**) cytoplasmic ADAR1 score (1) and nuclear ADAR1 score (3); (**D**) cytoplasmic ADAR1 score (2) and nuclear ADAR1 score (3); (**E**) cytoplasmic ADAR1 score (3) and nuclear ADAR1 score (5); (**F**) cytoplasmic ADAR1 score (3) and nuclear ADAR1 score (5). (**G**) Relationship with cytoplasmic and nuclear ADAR1 score of 92 patients with CC. (**H**) Kaplan–Meier curves for progression-free survival (PFS) rates of 92 patients with cervical cancer (CC) according to cytoplasmic ADAR1 status. (**I**) Kaplan–Meier curves for PFS rates of 92 patients with CC according to nuclear ADAR1 status. (**J**) Kaplan–Meier curves for PFS rates of 92 patients with CC according to the combination of cytoplasmic and nuclear ADAR1.

We investigated the relationship between ADAR1 expression and clinicopathological characteristics of the overall population. Patient characteristics are displayed in Table 1. For cytoplasmic ADAR1 staining, a score of 0 was observed in one case (1.1%), a score of 1 was observed in 30 cases (32.6%), a score of 2 was observed in 42 cases (45.6%), and a score of 3 was observed in 19 cases (20.7%). For nuclear ADAR1 staining, a score of 0 was observed in one case (1.1%), a score of 1 was observed in four cases (4.4%), a score of 2 was observed in 12 cases (13.0%), a score of 3 was observed in 21 cases (22.8%), a score of 4 was observed in 38 cases (41.3%), and a score of 5 was observed in 16 cases (17.4%) (Fig. 3A–F). Furthermore, cytoplasmic ADAR1 staining correlated with nuclear ADAR1 staining in patients with CC (R = 0.778, R^2^ = 605, *P* < 0.001) (Fig. 3G). Cytoplasmic and nuclear ADAR1 expression was also examined to determine their relationship with various clinicopathological parameters in patients with CC. However, no significant correlation was found between cytoplasmic and nuclear ADAR1 expression and various clinicopathological parameters, and only nuclear ADAR1 showed a tendency to correlate with myometrial invasion (*P* = 0.082, Table 2).

**Table1:** Patient and tumor characteristics.

Baseline characteristics	All patients	
Age at diagnosis, y	Mean, 46.5; range, 28–71	
	Numbers	(%)
Stage (FIGO 2018)		
I	56	60.9
II	19	20.6
III	17	18.5
Histology		
SCC	60	65.2
Adenocarcinoma	26	28.3
Adenosquamous carcinoma	6.5	
Tumor maximum size		
≤ 4 cm	68	73.9
> 4 cm	24	26.1
Myometrial invasion		
≤ 2/3	44	47.8
> 2/3	48	52.2
Parametrium invasion		
Absent	73	79.3
Present	19	20.7
Vagina invasion		
Absent	81	88
Present	11	12
LVS involvement		
Absent	36	39.1
Present	56	60.9
Lymph node metastasis		
Absent	75	81.5
Present	17	18.5

*SCC* squamous cell carcinoma; *LVS* lymph-vascular space.

**Table. 2:** Associations between cytoplasmic and nuclear ADAR1 and clinical factors of cervical cancer.

Variable	Numbers	Cytoplasmic ADAR1	*P*-value	Nuclear ADAR1	*P*-value
Histology			0.879		0.948
SCC	60	1.850 ± 0.570		3.516 ± 1.169	
Non-SCC	32	1.875 ± 0.564		3.500 ± 1.483	
Stage (FIGO 2018)			0.791		0.379
≤ I stage	56	1.875 ± 0.620		3.589 ± 1.519	
> I stage	36	1.833 ± 0.485		3.388 ± 0.873	
Tumor maximum size			0.604		0.789
≤ 4 cm	68	1.882 ± 0.582		3.529 ± 1.297	
> 4 cm	24	1.791 ± 0.519		3.458 ± 1.215	
Myometrial invasion			0.296		0.082
≤ 2/3	44	1.772 ± 0.598		3.295 ± 1.608	
> 2/3	48	1.937 ± 0.527		3.708 ± 0.891	
Parametrium invasion			0.915		0.651
Absent	73	1.863 ± 0.564		3.532 ± 1.391	
Present	19	1.842 ± 0.584		3.421 ± 0.812	
Vagina invasion			0.494		0.796
Absent	81	1.876 ± 0.584		3.518 ± 1.377	
Present	11	1.727 ± 0.418		3.454 ± 0.472	
LVS involvement			0.408		0.553
Absent	36	1.944 ± 0.739		3.416 ± 1.850	
Present	56	1.803 ± 0.451		3.571 ± 0.903	
Lymph node metastasis			0.536		0.198
Absent	75	1.880 ± 0.593		3.573 ± 1.356	
Present	17	1.764 ± 0.441		3.235 ± 0.816	

*ADAR* adenosine deaminase family acting on RNA; *SCC* squamous cell carcinoma; *LVS* lymph-vascular space.

*P < 0.05.

### The expression levels of cytoplasmic and nuclear ADAR1 could be used as prognostic markers for patients with CC

We next examined whether ADAR1 expression could be a predictive marker of progression-free survival (PFS) for patients with CC. In our cohort, the median PFS time for all patients was 46.0 months, and the follow-up period ranged from 1 to 126 months. At the last follow-up, 72 patients were alive with no evidence of the disease, nine patients had died of the disease, and 11 patients were alive with the disease. Overall, cytoplasmic and nuclear ADAR1 were most significantly found to be a prognostic factor for CC patients, based on PFS analysis using the log-rank test. The PFS curves of 92 patients with CC, according to their cytoplasmic and nuclear ADAR1 expression status, are shown in Fig. 3H–J. The PFS of patients exhibiting high cytoplasmic ADAR1 expression (score 2–3) was significantly longer than that of patients exhibiting low cytoplasmic ADAR1 expression (score 0–1) (*P* = 0.033; Fig. 3H). Furthermore, the PFS of patients exhibiting high nuclear ADAR1 expression (score 4–5) was significantly higher than that of patients exhibiting low nuclear ADAR1 expression (score 1–3) (*P* = 0.020; Fig. 3I). Based on cytoplasmic and nuclear ADAR1 expression, we divided the patients into four groups as follows: Group 1, low cytoplasmic and nuclear ADAR1 (n = 28); Group 2, high cytoplasmic ADAR1and low nuclear ADAR1 (n = 10); Group 3, low cytoplasmic ADAR1 and high nuclear ADAR1 (n = 3); and Group 4, high cytoplasmic and nuclear ADAR1 (n = 51). Patients with higher cytoplasmic and nuclear ADAR1 levels (group 4) showed poorer PFS than those in the other groups (*P* = 0.016; Fig. 3J).

Univariate and multivariate analyses were used to assess the correlation between clinical factors and PFS (Table 3). In univariate analysis, lymph-vascular space (LVS) involvement (*P* = 0.014), lymph node metastasis (*P* = 0.021), and higher cytoplasmic and nuclear ADAR1 expression (P = 0.017) were significantly associated with worse PFS. In multivariate analysis, LVS involvement (*P* = 0.038) and higher cytoplasmic and nuclear ADAR1 expression (*P* = 0.017) were significantly associated with worse PFS in patients with CC. The combination of high cytoplasmic and nuclear ADAR1 expression was found to be an independent predictor of prognosis for patients with CC.

**Table 3:** Prognostic factors for PFS selected by Cox’s univariate and multivariate analysis.

	Univariate analysis			Multivariate analysis		
	Exp(B)	95% CI	Cox’s test *P*-value	Exp(B)	95% CI	Cox’s test *P*-value
Histology (non-SCC)	1.897	0.788–4.563	0.153		-	
Stage (> II )	1.753	0.724–4.242	0.213		-	
Tumor size (maximum diameter: > 4 cm)	1.402	0.558–3.519	0.472		-	
Myometrial invasion (> 2/3)	2.369	0.909–6.171	0.077		-	
Parametrium invasion (Present)	0.823	0.292–2.320	0.713		-	
Vagina invasion (Present)	1.85	0.618–5.539	0.272		-	
LVS involvement (Present)	6.203	1.439–26.746	0.014*	4.932	1.096–22.189	0.038*
Lymph node metastasis (Present)	2.867	1.169–7.027	0.021*	2.038	0.809–5.132	0.131
High cytoplasmic ADAR1 and high nuclear ADAR1	3.827	1.724–11.496	0.017*	3.796	1.265–11.384	0.017*

*PFS* progression-free survival; *SCC* squamous cell carcinoma; *LVS* lymph-vascular space; *ADAR* adenosine deaminase family Acting on RNA.

## Discussion

CC is the most important manifestation of genital HPV infections. HPV viruses encode the E6 and E7 oncogenes, which are essential for malignant transformation and maintenance of the malignant phenotype of CC. HPV infection has been associated with the suppression of immune factors, including cytokines and chemokines, which results in the evasion of immune detection. Genetic polymorphisms in inflammatory pathways constitute a risk factor for CC development with HPV infections^29^.

A-to-I RNA editing is catalyzed by adenosine deaminase ADAR enzymes that bind and edit dsRNA. A-to-I RNA editing of the mRNA encoding oncogenes can alter tumor characteristics to promote a more aggressive phenotype. ADAR1 regulates innate immunity in response to viral infections, such as HPV^30^. ADAR1 has two isoforms, the shorter and constitutive ADAR1 p110 and full-length ADAR1 p150^13^. It has been proven that ADAR1 p150 is located in the cytoplasm and nucleus, whereas ADAR1 p110 is mainly expressed in the nucleus^14^. The ADAR1 p150 isoform is expressed at low levels basally compared with the p110 isoform but can be induced in response to a range of stimuli, such as infection with a dsRNA virus that induces an IFN response. ADARs are also involved in cancer immune recognition, mainly owing to the IFN response in various cancer types^31,32^. Chen et al. reported that high ADAR1 expression is significantly associated with the survival of patients with squamous cell carcinoma (SCC)^33^. However, there are no reports on the mechanism underlying the effects of ADAR1, and there are no such studies concerning patients with CC, including those with non-SCC. The biological functions and inhibitory effects of ADAR1 knockdown were investigated in HPV-positive and HPV-negative CC cell lines. Furthermore, ADAR1 was examined to clarify its correlation with clinicopathological parameters and prognosis in patients with CC, including non-SCC patients.

ADAR1 suppresses innate immunity primarily through RIG-I-like receptor (RLR)-initiated cytosolic dsRNA-sensing signaling pathways, including MDA5 and RIG-I^24,25^. RIG-I, MDA-5, and PKR induce apoptosis^26,27^. Similarly, activated ZBP1 can induce necroptosis^28^. To trace the steps involved in apoptosis and necroptosis, we evaluated the activation of MLKL, Caspase3, Caspase7, Caspase8, Bak, and Bax. The knockdown of ADAR1 led to increased ZBP1, MDA-5, RIG-I, PKR, IRF3, IRF-7, MLKL, Caspase3, Caspase7, Caspase8, Bak, and Bax expression, which in turn induced apoptosis and necroptosis in HPV-positive or HPV-negative CC cells. These results indicate that HPV exerts growth suppressive effects by effectively suppressing apoptosis and necroptosis in CC based on ADAR1 knockdown.

ADAR1 is overexpressed in hepatocellular carcinoma, gastric cancer, colorectal cancer, and endometrial cancer. Its overexpression is correlated with clinically aggressive behavior and patient survival^20–23^. Patients with high cytoplasmic and nuclear ADAR1 expression were found to have a significantly worse prognosis than patients exhibiting low cytoplasmic and nuclear ADAR1 expression. The combination of high cytoplasmic and nuclear ADAR1 expression was determined to be a factor associated with worse prognosis in patients with CC. Furthermore, multivariate analysis indicated that a combination of higher cytoplasmic and nuclear ADAR1 expression is an independent predictor of worse PFS in CC. Therefore, not only the ADAR1p150 isoform, but also the ADAR1p110 isoform, greatly influences the prognosis of CC patients.

The limitations of this study are as follows: first, it was a single-center study, and second, it was a retrospective analysis. Large-scale prospective studies are required to further ascertain the role and clinical significance of ADAR1 in CC. In summary, this study revealed the critical role of ADAR1 in patients with CC. ADAR1 increases the malignant potential of CC by inhibiting apoptosis and necroptosis. The present findings suggest that ADAR1 could be a new therapeutic target for CC. Furthermore, the combination of cytoplasmic and nuclear ADAR1 expression provides a better prognostic marker for CC.

## Material and methods

### Cell culture and siRNA transfection

HeLa cell lines were obtained from the Japanese Collection of Research Bioresources (JCRB) Cell Bank. SiHa cells were obtained from the American Type Culture Collection (ATCC). The HPV16-type SiHa, HPV18-type HeLa, and non-HPV-type Yumoto cell lines were maintained in Dulbecco’s Modified Eagle’s Medium (Life Technologies, CA, USA), supplemented with 10% fetal bovine serum. Cell lines were maintained in a humidified incubator containing 5% CO_2_ at 37 °C. Cells were used for functional experiments within 3 months of passaging post-receipt. The SiHa, HeLa, and Yumoto cell lines were trypsinized and plated in culture dishes. At ~ 50% confluency, the cell lines were transfected with an annealed ADAR1 siRNA (siADAR1, sc-37657; Santa Cruz Biotechnology, TX, USA), control siRNA (siControl, sc-37007; Santa Cruz Biotechnology, TX, USA), or an empty vector (mock) for gene silencing (final concentration, 100 nmol/L) using an siRNA transfection reagent (sc-29528; Santa Cruz Biotechnology, TX, USA).

### RNA isolation and real-time quantitative PCR analyses

Total RNA was isolated from CC cell lines using RNeasy Lipid Tissue Mini Kit (QIAGEN, Hilden, Germany). The iTaq Universal SYBR Green OneStep Kit and MiniOpticon Real-Time PCR System (Bio-Rad, CA, USA) were used for gene expression analysis via real-time quantitative PCR. *GAPDH* was used as a normalization control. Primer sequences for ADAR1, ADAR1p110, ADAR1p150, ZBP1/DLM-1/DAI (ZBP1), MDA5, RIG-I, PKR, IRF3, IRF7, MLKL, Caspase3, Caspase7, Caspase8, Bak, Bax, and GAPDH are presented in Supplementary Table 1.

### Apoptosis and necroptosis assay

Cell viability was analyzed using SYTO 10 (green fluorescence) and DEAD red (ethidium homodimer-2) nucleic acid stains (Live/Dead® reduced biohazard viability/cytotoxicity kit; Invitrogen, MA, USA). Briefly, cells were transfected with mock conditions, control siRNA, or ADAR1 siRNA for 48 h and then incubated with SYTO 10 and DEAD red nucleic acid stain for 15 min. Cell fluorescence was observed using a fluorescence microscope (Olympus, Tokyo, Japan). Apoptosis and necroptosis were measured through staining with FITC-conjugated annexin V using the MEBCYTO Apoptosis kit (MBL International Corp., MA, USA). Furthermore, apoptosis and necroptosis were analyzed using a FACS cytometer. Apoptosis and necroptosis were analyzed using a combination of imaging flow cytometry and classical annexin V/PI staining, as published previously^34^.

### MTS assay

The effect of ADAR1 on cell proliferation was evaluated using an MTS assay (Promega, WI, USA). Cells were seeded into 96-well plates and cultured until the cell density reached 3 × 10^3^ cells/well. The cells were then transiently transfected with control or ADAR1 siRNA for 48 h. After incubation with MTS for 1 h, absorbance was measured at a wavelength of 490 nm using an ELISA plate reader (Bio-Rad, CA, USA).

### Patients and tissue specimens

The institutional ethics committee approved the study at Okayama University (Approval number: 2112-014). Informed consent was obtained from all participants. All procedures were performed according to relevant ethical standards and institutional ethics committee regulations. In total, 92 patients with CC were treated at Okayama University Hospital from January 2008 to December 2017. Patients who had received chemotherapy and radiation before surgery were excluded from the study. All patients underwent radical hysterectomy and pelvic lymphadenectomy, with or without bilateral salpingo-oophorectomy. Pelvic lymph node dissection included the right and left common iliac nodes, external and internal iliac nodes, and supra-inguinal, obturator, sacral, and parametrial nodal chains. Adjuvant therapy was administered in accordance with the guidelines of the Japanese Society of Gynecologic Oncology (JSGO). Concurrent chemoradiation therapy consisted of external irradiation (50 Gy administered in 25 fractions over 5 weeks; 4-field box technique), high-dose intracavitary brachytherapy (24 Gy/four times), and concurrent chemotherapy (cisplatin 40 mg/m^2^ infusion weekly for six cycles). Following primary treatment, the patients underwent follow-up examinations approximately every 1–2 months for the first 6 months and subsequently every 3 months for the next 2 years. The Institutional Review Board of Okayama University Hospital approved this study protocol. Informed consent was obtained from all patients.

### Immunohistochemistry for ADAR1

Formalin-fixed paraffin-embedded (FFPE) blocks of representative primary tumor tissue were prepared. All FFPE specimens were cut into 4 μm-thick slices. FFPE sections were deparaffinized with xylene and rehydrated using an ethanol gradient. Endogenous peroxidase activity was blocked with H_2_O_2_, followed by antigen retrieval using citrate buffer at 98 °C for 20 min. Next, the slides were incubated overnight with an anti-ADAR1 antibody at a 1:100 dilution (Abcam, Cambridge, MA, USA). The sections were then incubated with biotinylated secondary antibodies (VECTASTAIN® ABC Kit, Vector Laboratories, Burlingame, CA, USA). Specific antigen–antibody reactions were visualized using diaminobenzidine tetrahydrochloride. Hematoxylin was used for cytoplasmic and nuclear counterstaining. The following cytoplasmic scales were used for staining degrees: 0, no staining; 1, weak staining; 2, moderate staining; and 3, strong staining. The proportion (%) of cells with positive nuclear staining was scored from 0 to 5 as follows: 0, 0–4%; 1, 5–19%; 2, 20–39%; 3, 40–59%; 4, 60–79%; 5, 80–100%). Quantification was performed for both cytoplasmic and the nuclear levels. Scores were first categorized as low (cytoplasm: 0–1; nucleus:0–1–2–3) and high (cytoplasm: 2–3; nucleus:4–5) based on ADAR1 staining intensity/percent positive expression. A second score was used to integrate information from cytoplasmic and nuclear expression, measured three times by two independent investigators blinded to the nature of the specimens and antibodies used.

### Statistical analysis

Statistical analyses were performed using the Mann–Whitney *U*-test for comparisons with controls and one-factor ANOVA followed by Fisher’s protected least significance difference test for all pairwise comparisons. Survival rates were calculated using the Kaplan–Meier method, and differences between the survival curves were examined using the log-rank test. The analyses were performed using StatView software (version 5.0; Abacus Concepts, Berkeley, CA, USA). Differences were considered statistically significant at *P* < 0.05.

### Ethics approval and consent to participate

Informed consent was obtained from each patient, and the institutional review board (The Ethics Committee of the Okayama University Graduate School of Medicine, Dentistry and Pharmaceutical Sciences and Okayama University Hospital) approved the study (2112-014).

## Supplementary Information

Supplementary Information.

## Data Availability

All data generated or analyzed during this study are included in this published article.
